# Chemically Induced Models of Parkinson’s Disease: History and Perspectives for the Involvement of Ferroptosis

**DOI:** 10.3389/fncel.2020.581191

**Published:** 2020-12-23

**Authors:** Shuheng Wen, Toshihiko Aki, Kana Unuma, Koichi Uemura

**Affiliations:** Department of Forensic Medicine, Graduate School of Medical and Dental Sciences, Tokyo Medical and Dental University, Tokyo, Japan

**Keywords:** Parkinson’s disease, ferroptos, paraquat, rotenone, 6-OHDA, MPTP

## Abstract

Ferroptosis is a newly discovered form of necrotic cell death characterized by its dependency on iron and lipid peroxidation. Ferroptosis has attracted much attention recently in the area of neurodegeneration since the involvement of ferroptosis in Parkinson’s disease (PD), a major neurodegenerative disease, has been indicated using animal models. Although PD is associated with both genetic and environmental factors, sporadic forms of PD account for more than 90% of total PD. Following the importance of environmental factors, various neurotoxins are used as chemical inducers of PD both *in vivo* and *in vitro*. In contrast to other neurodegenerative diseases such as Alzheimer’s and Huntington’s diseases (AD and HD), many of the characteristics of PD can be reproduced *in vivo* by the use of specific neurotoxins. Given the indication of ferroptosis in PD pathology, several studies have been conducted to examine whether ferroptosis plays role in the loss of dopaminergic neurons in PD. However, there are still few reports showing an authentic form of ferroptosis in neuronal cells during exposure to the neurotoxins used as PD inducers. In this review article, we summarize the history of the uses of chemicals to create PD models *in vivo* and *in vitro*. Besides, we also survey recent reports examining the possible involvement of ferroptosis in chemical models of PD.

## Introduction

Parkinson’s disease (PD) is a chronic, progressive, and irreversible neurodegenerative disorder, first medically described by James Parkinson in 1817 (Parkinson, 2002). As the second most common neurological disease, approximately 6,200,000 people around the world were suffering from PD in 2015, and the prospective number of PD patients keeps growing (Vos et al., 2016; Marras et al., 2018). However, the entire and precise etiology of PD still needs to be illuminated. Epidemiological evidence indicates both gene susceptibility and environmental factors could increase the risk of PD, with aging as the most vital risk factor (Collier et al., 2017). Sex difference also has been concerned as an important risk factor for PD, which is due to the predominant role of estrogens. Briefly, males have a risk of developing PD twice more often than females (Baldereschi et al., 2000), while females have a higher mortality rate (Larsson et al., 2018). Many genetic loci and mutations have been determined that can facilitate PD (Spatola and Wider, 2014). Sex-dependent mutations in genes like (Bakshi et al., 2019), *GBA1* (Bakshi et al., 2019), and *GADPH* (Ping et al., 2018) are correlated with the sex difference of PD. Certain genes are confirmed to be associated with the familial occurrence of PD by neuropathological examination and family-based genome-wide association studies (GWAS). For example, *α-synuclein* (*SNCA*), which might be involved in neuronal functions through facilitating SNARE-dependent vesicle fusion process, has been shown mutated in familial PD cases; point mutations in the *SNCA* gene such as A53T (Polymeropoulos et al., 1997), A30P (Krüger et al., 1998), and G51D (Lesage et al., 2013) could result in early-onset PD among family members. A GWAS study in the Japanese PD cohort revealed that *SNCA* is a major PD risk gene across the population (Simon-Sanchez et al., 2009). The microtubule-associated protein Tau (*MAPT*) gene, which is involved in stabilization of cytoskeleton and subsequent facilitation of the transport of neurotransmitters, has also been found as a locus associated with genetic risk for PD (Satake et al., 2009). *Parkin* (Kitada et al., 1998), and *PINK1* (Valente et al., 2004), both of which are involved in familial PD, are genes involved in mitophagy; upon mitochondrial damage, PINK1 accumulates on the mitochondrial outer membrane to attract E3 ubiquitin ligase parkin for clearance of damaged mitochondrial through autophagy (Pickrell and Youle, 2015).

Although PD can be inherited, it is more likely a sporadic disease because fewer than 10% of PD cases have an indisputable family history (Thomas and Beal, 2007). This means environmental factors appear to have more influence on the pathogenesis of PD. Adequate evidence has shown that chronic exposure to pesticides such as paraquat (PQ) and rotenone, as well as environmental pollutants such as preservatives and heavy metals, may raise the risk of PD (Semchuk et al., 1992; Seidler et al., 1996; Gorell et al., 1998; Tanner et al., 2011). Studies have also identified the ingestion of dairy products (Nicoletti et al., 2010), alcohol addiction (Nicoletti et al., 2010; Eriksson et al., 2013), and dependence on recreational drugs such as methamphetamine (Callaghan et al., 2010; Curtin et al., 2015) as possibly leading to a higher risk of PD.

The clinical diagnosis of PD is based mainly on the presence of associated and exclusive symptoms, medical history, as well as the efficacy of levodopa. PD symptoms are categorized as motor and non-motor symptoms in clinical practice. The classic motor symptoms applied to diagnose PD patients include rest tremor, muscular rigidity, bradykinesia, and postural impairment (Gibb and Lees, 1988). On the other hand, non-motor symptoms such as sleep disruption, depression, and constipation could start to bother PD patients in the early stages of PD even before diagnosis. These along with other disorders such as anxiety, dysphagia, early cognitive dysfunction, dementia, and hallucination represent the common non-motor symptoms of PD (Martinez-Martin et al., 2007; Barone et al., 2009; Schapira et al., 2017). Of note, males and females show distinctive clinical features in PD in addition to susceptibility and mortality (Georgiev et al., 2017). Most motor symptoms emerge later in females (Baba et al., 2005), while most non-motor symptoms tend to be more severe and common in them (Martinez-Martin et al., 2012). The hallmark of the pathophysiological changes in the PD brain is the severely impaired dopaminergic neurons in the substantia nigra, which is the cause of most of the main symptoms of PD. Lewy bodies in the remaining neurons of substantia nigra and other affected brain regions provide the most discriminating pathological observation in PD (Hassler et al., 1960; Hughes et al., 1992; Baba et al., 1998; Goedert, 2001).

## Brain Pathophysiology of PD

Clinical researchers consider the pathological progress of PD in the brain initiates in the lower structures of the brainstem, followed by a caudal-to-rostral pattern as well as the involvement of the cortico-basal ganglia-cerebellar pathways. It applies an ascending course that gradually affects the midbrain, especially the substantia nigra, and spreads from there to impair the mesocortex and neocortex. As the pathologic progress continues, the neurodegeneration of formerly impaired regions is also aggravated (Braak et al., 2003). The lesions in the brainstem will trigger the reorganization within the cortico-basal ganglia-cerebellar pathways, and recruiting alternative pathways to compensate for the initial impairment at the early stage (Quartarone et al., 2020). The response of the cerebellum finally becomes maladaptive and induces the clinical motor symptoms of PD patients (Wu and Hallett, 2013; Caligiore et al., 2017). Postmortem and *in vivo* studies also linked the damage to the noradrenergic neurons in the locus coeruleus in the early stage of PD and the dopaminergic neurons in the substantia nigra pars compacta with the contribution of the motor deficits seen in PD patients (Fearnley and Lees, 1991; Patt and Gerhard, 1993; Shin et al., 2014). Nevertheless, due to the compensative mechanisms of the basal ganglia, thalamic nucleus, and the cerebellum, the clinical symptoms of motor deficits do not appear until 50%–70% of the dopaminergic neurons in substantial nigra are impaired and the dopamine levels in the striatum are depleted by approximately 80% (George et al., 2009; Obeso et al., 2010; Alberio et al., 2012). By contrast, understanding of the pathophysiological mechanism responsible for the non-motor symptoms remains limited, although degenerations of the dopaminergic and nondopaminergic systems are suggested to be involved (Ahlskog, 2005). Neuroinflammatory responses are also implicated in the non-motor symptoms of PD since increased levels of inflammatory markers in the cerebrospinal fluid are significantly associated with more severe symptoms of depression, anxiety, fatigue, and cognition in PD patients (Lindqvist et al., 2013).

Not only do the etiology and pathophysiology of the non-motor symptoms of PD remain unclear, but the precise mechanism for the death of neurons in PD is also uncertain. Various interpretations have been made to determine whether neuronal death in PD is simply physiological apoptosis, a pathologic process, or even more complicated. *In vitro* and *in vivo* studies as well as postmortem observations have shown that intrinsic caspase-dependent apoptosis participates in dopaminergic cell death in PD (Hartmann et al., 2000; Viswanath et al., 2001). Intrinsic caspase-independent apoptosis is also suspected of being involved in neuronal death in PD; cytochrome *c*-independent apoptosis through endonuclease G is implicated in α-synuclein cytotoxicity (Li et al., 2001; Büttner et al., 2013). On the other hand, robust evidence of activated microglia in the substantia nigra found on postmortem examination of PD brain supports the view that neuroinflammation is involved in dopaminergic neuronal death in PD (McGeer et al., 1988; Banati et al., 1998), which is characterized by activated resident microglia and an absence of reactive astrocytosis (Mirza et al., 2000).

Lewy bodies have also gained a lot of attention as to their specific distribution in neurons, and increases in their amount are consistent with both PD progression and neurodegeneration (Bethlem and Den Hartog Jager, 1960; Qualman et al., 1984; Gibb and Lees, 1988; Braak et al., 2003, 2004). Lewy bodies are weakly acidophilic round inclusion bodies formed mainly by aggregation of a presynaptic protein, α-synuclein (Spillantini et al., 1997). α-Synuclein is believed to play a pivotal role in the initiation and progression of inflammation in PD. Studies indicate that the aggregation of α-synuclein might be responsible for neuronal death in PD. *In vitro* and *in vivo* studies have determined the toxicity of α-synuclein oligomers to membranes (Conway et al., 2000; Danzer et al., 2007; Karpinar et al., 2009; Winner et al., 2011). Postmortem studies have revealed the aggregation of α-synuclein in neurons around pigment-associated lipids under oxidative conditions, and this aggregation may participate in neuronal death in PD (Halliday et al., 2005). Inspection of the brain and cerebrospinal fluid of PD patients by sensitive sandwich enzyme immunoassay found an apparently higher level of tumor necrosis factor α (TNFα), which links extrinsic apoptosis with dopaminergic neuronal death in PD (Mogi et al., 1994). It is worth noticing that α-synuclein and its mutations are also linked to these cell death mechanisms as essential inducers or mediators (Martin et al., 2006; Su et al., 2009; Büttner et al., 2013).

## Cellular Pathophysiology of PD

**Figure 1:**
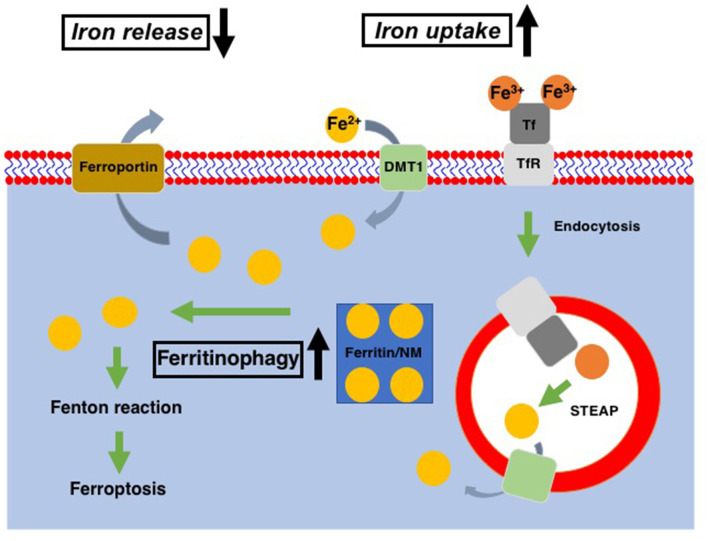
Cellular iron metabolism. Ferric iron (Fe^3+^) is transported into cells *via* binding to transferrin receptor (TfR) and subsequent endocytosis, while ferrous iron (Fe^2+^) enters cells through divalent metal transporter1 (DMT1). Within the endosomes, Fe^3+^ is reduced to Fe^2+^ through the action of the six-transmembrane epithelial antigen of the prostate (STEAP, metalloreductase) and released into the cytosol *via* DMT1. Ferritin is served as the intracellular reservoir of iron. In DA neurons, neuromelanin (NM) works as the iron storage protein instead of ferritin. Fe^2+^ stored in ferritin/NM is released into the cytosol as the cytosolic labile iron pool *via* ferritinophagy. Excess in the cytosolic labile iron pool leads to the export of excess iron *via* ferroportin. In Parkinson’s disease (PD) patients as well as PD models, cellular iron uptake and release were increased and decreased, respectively, in DA neurons. Also, ferritinophagy is upregulated in the PD model. All of these contribute to the increase of cytoplasmic labile iron pool, which leads to an increase of ferroptosis susceptibility of DA neurons in PD.

As the comprehension of cellular physiology has deepened, several mechanisms have also been assumed to take part in the neuronal death in PD. The morphological assessment has linked autophagy to the substantia nigra dopaminergic neuronal death of PD patients (Anglade et al., 1997). Increased reactive oxygen species derived from mitochondria have been indicated to cause lysosomal dysfunction, which induces the later accumulation of autophagosomes in the PD brain (Dehay et al., 2010). Studies implied that familial PD-associated genetic mutations, such as in DJ-1 and ATP13A2, might impair autophagy and contribute to the neuronal death in PD (Krebiehl et al., 2010; Dehay et al., 2012; Gusdon et al., 2012). An *in vitro* study suggested that necroptosis is involved in the pathogenesis of PD through excitotoxicity, the accumulation of intracellular Ca^2+^ or TNFα, although its contribution to PD pathology is uncertain (Beal, 1998; Li et al., 2008; Edwards et al., 2011). Iron has also been suspected as a culprit in the increased oxidative stress and lesions of dopaminergic neurons because of its potent reductive property (Jenner et al., 1992). When it comes to the sex differences in PD, female dopaminergic neuronal cells are less vulnerable to degenerative factors than male neurons, mainly due to the impact of estrogens. That is because estrogens not only have anti-inflammatory properties but also preserve lipid balance in neuronal membrane microdomains (Marin and Diaz, 2018). Estrogens and their selective receptor modulators have been shown to reduce the apoptosis, oxidative stress, mitochondrial membrane depolarization, and Ca^2+^ influx of neurons (Yazğan and Naziroğlu, 2017; Chen et al., 2019). Also, female neurons have higher electron transport chain activity and greater functional capacities compared with male neurons, which result in lower oxidative stress (Escames et al., 2013; Harish et al., 2013; Gaignard et al., 2015). Iron accumulation in DA neurons (summarized graphically in Figure 1) has reported repeatedly in PD patients as well as model animals, suggesting dysfunction of iron metabolism in PD (Jiang et al., 2017; Santiago and Potashkin, 2017; Moreau et al., 2018). As a newly discovered mechanism of iron-induced cell death, ferroptosis is considered a significant candidate for the major death process of dopaminergic neuronal cells due to its close relationship with iron and lipid peroxidation (Dixon et al., 2012; Skouta et al., 2014; Do Van et al., 2016). Considering the properties of estrogens to maintain the lipid balance in neuronal membrane microdomains and to regulate the iron metabolism of neurons (Wang et al., 2015; Mariani et al., 2016), it is very likely that sex differences also affect the process of ferroptosis.

## Ferroptosis

**Figure 2:**
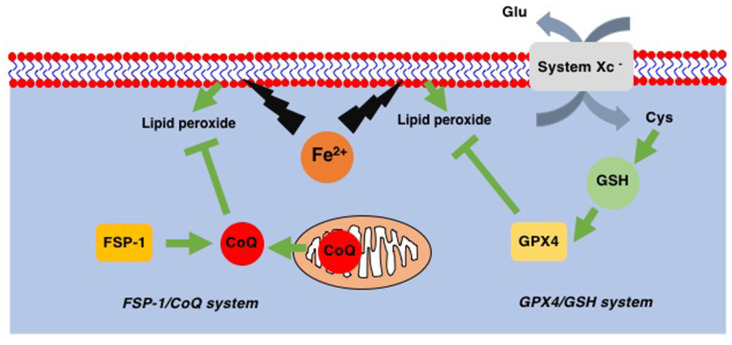
Two ferroptosis surveillance systems. Glutathione peroxidase-4 (GPX4) belongs to the glutathione (GSH)-dependent peroxidase family of proteins and is the only protein in this family that can reduce lipid peroxides. System Xc^−^ is the cystine-glutamate exchange system through which intracellular GSH levels are maintained. In addition to this GPX4/GSH system, ferroptosis suppressor protein-1 (FSP-1), a CoQ oxidoreductase that reduces coenzyme Q_10_ (CoQ) to regenerate as a radical scavenger, has been found. This FSP-1/CoQ system is another system to eliminate lipid peroxides from healthy cells.

Ferroptosis is a recently identified form of cell death that takes place in a caspase-independent but regulated manner. By its independence from caspase, ferroptosis is morphologically categorized as necrosis rather than apoptosis (Dixon et al., 2012). The most striking feature of ferroptosis is its association with lipid peroxidation (Figure 2; Yang et al., 2014). In contrast to the relatively lower contribution of lipids to other forms of cell death such as apoptosis, pyroptosis, and necroptosis, ferroptosis relies exclusively on lipid peroxidation. Therefore, the loss of glutathione peroxidase 4 (GPX4), which is the only enzyme in the GPX family with the ability to reduce peroxidized lipids (Figure 2; Brigelius-Flohé and Maiorino, 2013), results in ferroptosis (Yang et al., 2014). Since GPX4 utilizes reduced glutathione (GSH) to reduce lipids, decreased levels of intracellular GSH also lead to ferroptosis through the inability of GPX4 to reduce peroxidized lipids. Intracellular GSH levels are maintained *via* the incorporation of extracellular cystine, an oxidized form of cysteine. The incorporation of cystine through system Xc^−^ on the plasma membrane results in a subsequent increase in intracellular cysteine, a precursor of GSH (Figure 2). This GSH/GPX4 system works as the main ferroptosis surveillance system in healthy cells, and, therefore, the inhibitions of system Xc^−^ by erastin and GPX4 by RSL3 result in ferroptosis (Dixon et al., 2012).

In addition to the GSH/GPX4 system, another system to avoid the accumulation of peroxidized lipids has been discovered. Mitochondrial CoQ oxidoreductase has been identified as a protein that can protect cells from ferroptosis when GPX4 is absent (Bersuker et al., 2019; Doll et al., 2019). This enzyme was therefore renamed ferroptosis suppressor protein-1 (FSP-1; Figure 2). It is known that CoQ can reduce peroxidized lipids (Frei et al., 1990). Thus, these reports not only reveal a novel ferroptosis surveillance system, the CoQ/FSP-1 system but also solve the long-existing mystery as to why CoQ (CoenzymeQ, ubiquinone), an essential component of the mitochondrial electron transfer chain (ETC), is also located in extramitochondrial spaces where CoQ function was not known (Morré and Morré, 2011).

Lipid peroxidation during ferroptosis proceeds as follows: (1) the initial reaction occurs between ROS (e.g., hydroxyl radicals), that seem to be derived from NADPH oxidase rather than mitochondrial ETC, at least under some circumstances (Dixon et al., 2012), and hydrogen atoms in unsaturated lipids, typically polyunsaturated fatty acids (PUFAs). During ferroptosis, PUFAs should be incorporated into the membrane in a phospholipid (PL)-conjugated form (PUFA-PLs) through the action of acyl-CoA synthetase long chain-4 (ACSL4) and lysophosphatidylcholine acyltransferase-3 (LPCAT3; Dixon et al., 2015; Doll et al., 2017; Kagan et al., 2017). Lipid peroxides are generated *via* enzymatic oxygenation by 15-lipoxygenase (LOX15; Dixon et al., 2015) and/or the iron-dependent non-enzymatic Fenton reaction (Winterbourn, 1995). The discovery of small chemical inducers and inhibitors of ferroptosis has supported progress in this new research area. In addition to the availability of authentic ferroptosis inducers such as erastin (system Xc^−^ inhibitor) and RSL3 (GPX4 inhibitor), there are several reliable ferroptosis inhibitors. For example, ferrostatin-1 (Fer-1) and liproxstatin-1 (lip-1) are lipophilic radical scavenging molecules that are thought to eliminate lipid ROS (Dixon et al., 2012; Zilka et al., 2017), while deferoxamine (DFO) is a well-known iron chelator. Although the mechanisms for the initiation and progression of ferroptosis have been revealed, the way in which lipid peroxides cause plasma membrane rupture remains unknown. A report showed that peroxidized lipids alter membrane fluidity in liposomes (Borst et al., 2000), although whether or not peroxidized lipids or their derivatives can cause membrane rupture has not been elucidated.

## Chemicals Used to Create PD Model

**Figure 3:**
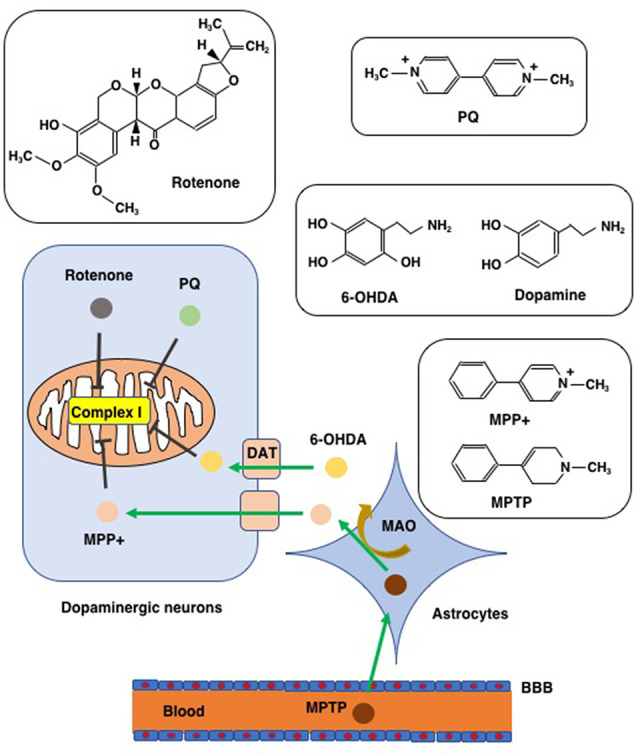
Chemicals used to create PD models. Chemical structures of rotenone, paraquat (PQ), MPTP/MPP+, and 6-hydroxydopamine (6-OHDA), are shown along with that of dopamine for comparison to 6-OHDA. MTPT can penetrate the blood-brain barrier (BBB) and enter into astrocytes where MPTP is converted to MPP+ *via* the action of monoamine oxygenase (MAO). MPP+ enters into dopaminergic neurons through the dopamine transporter (DAT). Due to its structural similarity to dopamine, 6-OHDA can also enter dopaminergic neurons *via* DAT. Rotenone, PQ, MPP+, and 6-OHDA all inhibit complex I of the mitochondrial respiratory chain.

To clarify the pathophysiological process of PD and to evaluate the efficiency of PD-targeted medicines, sophisticated models have been developed that use a panel of chemicals to reproduce and imitate the neurodegeneration process of PD. We introduce four chemicals (PQ, rotenone, 6-OHDA, and MPTP/MPP+) that were applied to create the PD model for their capability to cause PD-like neuron impairments and symptoms (Figure 3). The ability of these chemicals to induce ferroptosis will also be discussed with special attention paid to their effects in SH-SY5Y human neuroblastoma cells since these cells possess properties of dopaminergic neurons, and, therefore, have a long history of use as an *in vitro* model in studies on PD (Cheung et al., 2009). Early studies using SH-SY5Y cells as an *in vitro* model of chemically-induced PD showed that apoptosis is the main mode of cell death induced by PQ (Klintworth et al., 2007; Yang et al., 2009), rotenone (Kitamura et al., 2002; Klintworth et al., 2007), 6-OHDA (von Coelln et al., 2001), and MPP+ (Itano and Nomura, 1995; Sheehan et al., 1997). This might be, at least in part, to the fact that apoptosis was the only mode of cell death that could be examined using marker proteins (caspases and their substrates) as well as electron and fluorescence microscopy when the studies were performed. However, recent research progress has shown that SH-SY5Y cell death due to PD-inducing chemicals involves forms of cell death other than apoptosis.

Unfortunately, a perfect model that captures every characteristic and detail of PD does not exist at present. Due to various limitations, such as the stability of models, properties of chemicals, and differences between experimental animal species and humans, models currently in use can only represent one or several views of the pathophysiological progress of PD (Miller, 2007; Berry et al., 2010). Although it is still difficult to fully understand the disease progress, it is possible to choose the most suitable model for a particular study aim and still achieve satisfying and indicative results.

### Paraquat

Paraquat (1,1′-dimethyl-4,4′-bipyridnium, PQ), an important member of the bipyridyl family of broad-spectrum compounds, was developed in the early 1960s by the Syngenta company as a nonselective quaternary ammonium herbicide. PQ is one of the world’s most commonly used weed killers. Although the use of PQ is banned by many countries due to its fatal toxicity towards humans, its manufacture and exportation are controversially permitted (Bastias-Candia et al., 2019). In America and Japan, and many developing countries that allow its use, PQ is still widely sprayed on fields with worrying consequences. The worldwide annual incidence of PQ poisoning is approximately 38/1,000,000, with extremely high mortality rates that vary from 54 to 74% (Weng et al., 2012; Ko et al., 2017; Elenga et al., 2018). Most PQ poisoning cases are related to suicide (Gawarammana and Dawson, 2010).

#### Toxicity of Paraquat

Soon after PQ was put into practical use, it’s poisoning in humans was reported (Bullivant, 1966). Acute exposure of humans to PQ causes severe and irreversible damage to the lungs, kidneys, and liver, which eventually leads to death even if the dose is not massive. This is mainly because PQ rapidly accumulates and persists in these organs during its distribution in the human body (Sharp et al., 1972; Rose et al., 1976; Houzé et al., 1990). With poor clearance and metabolism, PQ can persist in the plasma and urine for weeks and months after poisoning, thereby inducing continuous damage (Houzé et al., 1990). The cause of death in PQ poisoning cases is mostly pulmonary fibrosis. PQ is taken up into the lungs with a higher concentration and longer persistence than other organs soon after acute exposure (Rose et al., 1974; Houzé et al., 1990). In the lungs, PQ causes widespread edema, acute alveolitis, alveolar collapse, inflammatory reactions in the vascular endothelium, pulmonary congestion, hemorrhage, and fibrosis in the long term (Dearden et al., 1978, 1982; Gawarammana and Buckley, 2011). PQ also impairs the renal parenchyma, and causes ischemic or toxic acute tubular necrosis in the kidneys, which leads to acute kidney injuries (Bullivant, 1966; Oreopoulos et al., 1968; Fowler and Brooks, 1971; Kim et al., 2009). Congestion and hepatocellular injury can be observed after PQ accumulation in the liver (Matsumori et al., 1984). The clearance and detoxication of PQ depend on the kidneys and liver, so the reduction in their functions further contributes to the persistence of PQ in the body and increases its toxicity and mortality (Houzé et al., 1990).

**Figure 4:**
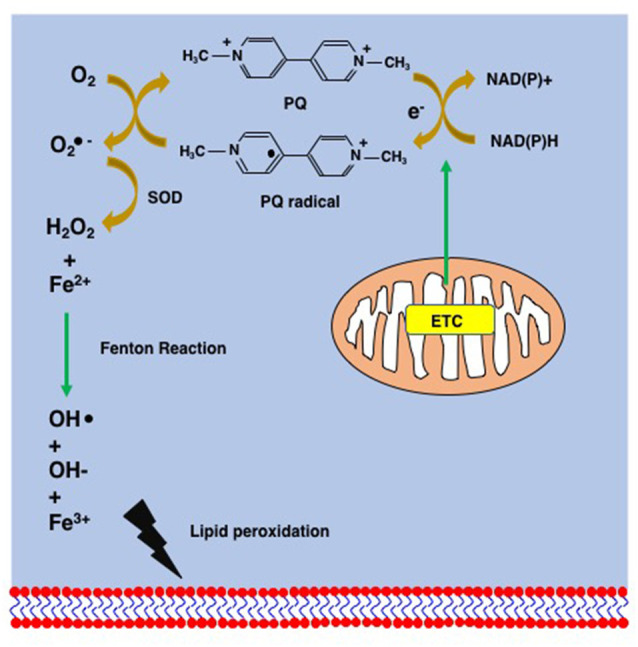
Supposed mechanism of lipid peroxidation by PQ. PQ becomes the PQ radical after taking an electron from the mitochondrial electron transfer chain (ETC). The PQ radical reacts with molecular oxygen and generates the superoxide anion (${\text{O}}_{2}^{-}$), which is finally converted to hydroxy peroxide (H_2_O_2_) *via* the action of superoxide dismutase (SOD). The Fenton chemistry between Fe^2+^ and H_2_O_2_ leads to the peroxidation of lipids.

The inherent toxicity of PQ is mainly due to its redox cycling in cells and to mitochondrial toxicity (Figures 3, 4). PQ is metabolized by cellular enzyme systems including the NADPH-cytochrome P450 reductase (Clejan and Cederbaum, 1989; Kelner and Bagnell, 1989) and NADPH oxidase (Bus and Gibson, 1984; Peng et al., 2009). During metabolism, redox cycling of PQ generates reactive oxygen species and initiates lipid peroxidation (Figure 4; Bus et al., 1976a,b; Adam et al., 1990; Castello et al., 2007). The increasing oxidative stress and lipid peroxidation induce apoptosis and contribute to PQ toxicity (Bus et al., 1976a; Kurisaki, 1985; Rio and Velez-Pardo, 2008; Yang and Tiffany-Castiglioni, 2008). PQ is principally reduced by Complex I in mitochondria, where PQ induces an increase in the Ca^2+^-dependent permeability of the mitochondrial membrane and forms superoxide. Both contribute to the mitochondrial toxicity of PQ by damaging the mitochondrial inner membrane and mitochondrial function (Costantini et al., 1995; Cocheme and Murphy, 2008; Gawarammana and Buckley, 2011).

#### Neurotoxicity of Paraquat

The overlap and similarities between the pathophysiology of PD and the neurotoxicity of PQ, such as increased oxidative stress and the aggregation of α-synuclein, make it natural for researchers to associate their relationship. Abundant and detailed evidence has demonstrated that chronic exposure to PQ increases the PD risk (Hertzman et al., 1990; Liou et al., 1997; Ossowska et al., 2005; Kamel et al., 2007; Tanner et al., 2011).

Because the concentration of PQ in the brain following acute exposure is low, the possibility of neurotoxicity by PQ was overlooked in earlier studies as it was thought that the blood-brain barrier (BBB) would impede PQ entry into the brain; rather, the presence of PQ in the brain was attributed to the cerebral circulatory system (Houzé et al., 1990; Naylor et al., 1995; Widdowson et al., 1996). However, cases of brain damage caused by PQ began to be reported (Grant et al., 1980; Hughes, 1988), and research then verified the ability of PQ to penetrate the BBB. The findings indicated that PQ penetrates the brain not through destruction of BBB function, but rather by a process mediated by the neutral amino acid transport system. PQ then penetrates striatal neurons in a Na^+^-dependent manner (Shimizu et al., 2001; McCormack and Di Monte, 2003). Chronic low-dose exposure to PQ demonstrates a more persistent and apparent PQ concentration with neurodegeneration in the brain as compared to one-time acute exposure (Prasad et al., 2007). Case reports showed that PQ induces brain edema, subarachnoid hemorrhage, neuroinflammation, and moderate neuronal damage (Grant et al., 1980; Hughes, 1988). Subsequent studies revealed that PQ produces selective lesions in the dopaminergic neurons of the substantia nigra (McCormack et al., 2002; Kang et al., 2009), and impairs noradrenergic neurons in the locus coeruleus (Fernagut et al., 2007; Sandstrom et al., 2017), solitary nucleus, and catecholaminergic neurons in the pituitary-adrenal system (Edmonds and Edwards, 1996), all of which subsequently decrease the dopamine concentration in the brain (Barbeau et al., 1985).

Studies have indicated that PQ dopaminergic neurotoxicity is associated with increased cellular oxidative stress and neuroinflammation, which induces α-synuclein aggregation and impairs neurons (Manning-Bog et al., 2002; Fernagut et al., 2007; Mak et al., 2010). Under the generation of ROS and increased oxidative stress with or without inhibition of the antioxidative glutathione system of the substantia nigra, PQ elicits mitochondrial dysfunction and injury, and eventually causes neurons to embark on cell death mechanisms (Yang and Tiffany-Castiglioni, 2005; Doostzadeh et al., 2007; Cocheme and Murphy, 2008; Cristovao et al., 2009; Kang et al., 2009; Niso-Santano et al., 2010). Several signaling pathways are reported to be involved in this final act. The JNK signaling pathway is considered to be a direct mediator in PQ-induced neuronal apoptosis (Peng et al., 2004). The inhibition of the Wnt signaling pathway is related to the severe neurodegenerative progress induced by PQ, including demyelination (Inestrosa and Arenas, 2010). An *in vitro* study showed that apoptosis is involved in the oxidative stress-related dopaminergic neurotoxicity of PQ (Peng et al., 2004). The axonal guidance and Wnt/β-catenin signaling contribute to the loss of dopaminergic neurons and the increased α-synuclein, identified by transcriptome sequencing in the ventral midbrain and striatum of PQ treated mice (Gollamudi et al., 2012). Ferroptosis has also been reported to be associated with damage to the locus coeruleus noradrenergic neuronal degeneration, and the occurrence of non-motor symptoms such as learning and memory dysfunction, which take place in the early stage of PD (Hou et al., 2019b).

We have shown that is difficult to explain PQ toxicity towards SH-SY5Y cells by apoptosis, necroptosis, or ferroptosis (Hirayama et al., 2018). However, Hou et al. (2019b) demonstrated that PQ combined with maneb seems better at inducing PD-like progress, especially the pathological changes seen in the early stages of PD. Indeed, they also demonstrated that PQ plus maneb induces ferroptosis in SH-SY5Y cells (Hou et al., 2019a) and that this can be suppressed by ferrostatin-1, liproxstatin-1, or deferoxamine (Hou et al., 2019a). Furthermore, cell death could be accelerated by the addition of iron. GPX4 and GSH levels were also reduced, suggesting that SH-SY5Y cell death by PQ plus maneb is associated with many of the features of ferroptosis. The combined administration of PQ with maneb or its closely related chemical, mancozeb, has also been applied to study the effects of PQ on the nigrostriatal dopamine system and was found to reproduce PD symptoms better than PQ alone (Thiruchelvam et al., 2000a,b). Maneb is a carbamate fungicide that has been shown to cause mitochondrial damage (Zhang et al., 2003). Complex III is considered to be the main target of maneb (Zhang et al., 2003), in contrast to PQ toxicity towards complex I. Also, a recent study demonstrated that maneb has broad cellular effects including causing alterations in glycolysis (Anderson et al., 2018). Although the differences between the neurotoxic mechanisms of PQ and PQ plus maneb have not been elucidated in detail, these reports put PQ plus maneb as a strong candidate as a future model for not only PQ pathophysiology but also for the mechanisms of ferroptosis.

### Rotenone

Rotenone is a naturally occurring compound commonly used worldwide as an herbicide and insecticide. It is extracted from plants including *Lonchocarpus*, *Millettia pachycarpa*, and *Mundulea suberosa* (Clark, 1929). Rotenone was first used as a fish poison until its potential for insect control was noticed. The popularized application of rotenone in agriculture is due to its easy decomposition under sunlight, short half-life, and specific remarkable toxicity against insects. Though hardly any cases of rotenone toxicity to humans have been reported, rotenone ingestion can cause metabolic acidosis, respiratory dysfunction, neurological symptoms, and injuries to the cardiovascular system, liver, and brain (Wood et al., 2005; Chesneau et al., 2009; Patel, 2011). The neurotoxicity of rotenone to nigrostriatal dopaminergic neurons was first discovered in an *in vivo* study in 1985 (Heikkila et al., 1985). Unlike PQ, the high lipophilicity of rotenone allows it to penetrate the BBB and all types of cells without the involvement of a transport system and then impairs the brain. Evidence indicates that rotenone selectively impairs the striatum, globus pallidus, and nigrostriatal dopaminergic neurons (Ferrante et al., 1997; Betarbet et al., 2000).

The neurotoxicity of rotenone is mainly the result of its mitochondrial toxicity and mitotic inhibition. In neuronal cells, rotenone impairs mitochondrial energy metabolism by inhibiting complex I (Ferrante et al., 1997; Talpade et al., 2000; Schuler and Casida, 2001). Studies indicate that PD is positively associated with rotenone exposure (Spivey, 2011). The RNA sequencing analyses of rat enteric nervous cell PD model induced by rotenone showed that Mitogen-Activated Protein Kinase regulates the PD pathogenesis. The Toll-like receptor, Wnt, and Ras signaling pathways intensively participate in the neurotoxicity of rotenone, particularly in neurodegeneration and aggregation of α-synuclein (Guan et al., 2017). Rotenone induces PD-like motor symptoms such as muscular rigidity, bradykinesia, and the aggregation of α-synuclein (Betarbet et al., 2000; Greenamyre et al., 2010). However, early attempts to apply rotenone as a PD model found that high doses of rotenone induce widespread brain damage rather than specifically targeting the nigrostriatal system, which might interfere with the value of relevant studies (Heikkila et al., 1985; Ferrante et al., 1997; Rojas et al., 2009). Modified rotenone PD models using a low dose and chronic administration have shown promising outcomes with selective nigrostriatal neurodegeneration and positive aggregation of α-synuclein cytoplasmic inclusions (Betarbet et al., 2000; Sherer et al., 2003; Zhu et al., 2004; Inden et al., 2011). Although it seems to be a perfect model for PD studies, there are criticisms that the rotenone-induced PD model is difficult to reproduce and likely to cause mortality (Höglinger et al., 2003; Fleming et al., 2004; Lapointe et al., 2004; Zhu et al., 2004; Johnson and Bobrovskaya, 2015). These limitations might be explained by the heterogeneous distribution of rotenone in the brain (Talpade et al., 2000). It is worth mentioning that although rotenone-induced PD models have been widely used in PD studies, there almost no cases of rotenone-induced PD in humans. There might be two reasons for this scarcity. First, the short half-life and easy decomposition of rotenone decrease the risk of its exposure to humans. Second, after ingestion, the absorption of rotenone in the gastrointestinal tract is slow and incomplete, while it is effectively metabolized in the liver. These properties make it unlikely to enter the general circulation to cause bad outcomes unless the intake is excessive.

Many studies have demonstrated that rotenone induces apoptosis in SH-SY5Y cells. However, Kabiraj et al. (2015) have demonstrated that apoptotic SH-SY5Y cell death induced by rotenone can be suppressed by Fer-1. They also demonstrated that Fer-1 mitigates not only rotenone-induced apoptosis but also synuclein aggregation and ER stress. Although the mechanism of cell death seems to be apoptosis as demonstrated by the appearance of positive apoptosis markers such as PARP cleavage, the effectiveness of Fer-1 in reducing cellular damage may indicate that the rotenone-induced apoptosis of SH-SY5Y cells has some of the characteristics of ferroptosis.

### 6-OHDA

6-Hydroxydopamine (6-OHDA), a highly oxidizable dopamine analog, was first identified in 1959 (Tieu, 2011). Unlike more commonly used herbicides such as PQ, 6-OHDA is away from public attention and mainly applied in scientific research to build PD models. In the beginning, 6-OHDA was found to lessen noradrenaline concentrations in the brains of mice (Porter et al., 1965). Further studies recognized the potential of 6-OHDA in PD research because it can decrease the concentrations of dopamine and noradrenaline in the brains of neonatal rats (Breese and Traylor, 1972). Electron microscopic studies revealed that 6-OHDA selectively impairs sympathetic adrenergic nerve terminals (Tranzer and Thoenen, 1968, 1973). Besides impairing neurons, 6-OHDA also induces PD-like motor and non-motor symptoms in rats (Ungerstedt, 1968; Luthman et al., 1989; Branchi et al., 2008; Tadaiesky et al., 2008). These properties of inducing PD-like neurodegeneration and symptoms make 6-OHDA a popular choice for building PD models. The neurotoxicity of 6-OHDA is mainly related to oxidative stress. After administration, 6-OHDA is absorbed into neurons by dopaminergic and noradrenergic transporters due to its structural similarities to dopamine and noradrenaline (Luthman et al., 1989). In neuron cells, 6-OHDA generates ROS by oxidation and the Fenton reaction (Blum et al., 2001), and this increased intracellular oxidative stress leads to lipid peroxidation (Saner and Thoenen, 1971; Graham, 1978; Kumar et al., 1995). 6-OHDA has also been shown to impair the mitochondrial respiratory chain by inhibiting complex I (Glinka and Youdim, 1995). Furthermore, studies suggest that neuroinflammation is involved in the neurotoxicity of 6-OHDA (Stromberg et al., 1986). Consistently, the RNA sequencing analyses of the 6-OHDA-induced PD rat model identified differentially expressed genes like *IRF7, ISG15*, et al. that contribute critical roles in early neuroinflammatory response and neurodegeneration (Li et al., 2019). To obtain neurotoxic concentrations in the brain, 6-OHDA must be administrated by intracerebral injection because it hardly crosses the BBB (Sauer and Oertel, 1994). Meanwhile, the strategy of injection has a significant effect on the subsequent neurodegeneration (Agid et al., 1973).

6-OHDA has been reported inducing ferroptosis in an *in vitro* model using SH-SY5Y cells (Sun et al., 2020). In contrast, there is a report demonstrating that the non-oxidative form of dopamine can inhibit erastin-induced ferroptosis (Wang et al., 2016). Dopamine suppresses erastin-induced ferroptosis by inhibiting the degradation of GPX4 as well as dopamine receptors (Wang et al., 2016). Also, dopamine itself is a powerful anti-oxidant; the anti-oxidative effect of dopamine is stronger than that of α-tocopherol (Yen and Hsieh, 1997). Therefore, dopamine itself should be involved in the avoidance of ferroptosis through multiple means including anti-oxidative properties and suppression of GPX4 degradation, the latter of which also leads to an anti-oxidative effect in DA neurons.

### MPTP

1-methyl-4-phenyl-1,2,3,6-tetrahydropyridine (MPTP), a meperidine congener, was accidentally produced in the early 1980s during an impure synthesis of MPPP, which is an opioid analgesic drug (Davis et al., 1979; Langston et al., 1983). Instead of any psychostimulant effect, its neurodegenerative potential was recognized very soon. PD-like degeneration of nigrostriatal neurons and motor symptoms was found in humans who misused MPTP (Davis et al., 1979; Langston et al., 1983, 1999s). Since then, MPTP has been widely used to create a PD model for research. MPTP was found to be a prodrug for the neurotoxin MPP+ (1-methyl-4-phenylpyridinium). Because MPTP is a lipophilic compound, it can cross the BBB, and be taken up by astrocytes where it is converted to MPP+. The MPP+ is then released into the extracellular space and subsequently absorbed into dopaminergic neurons and terminals by dopaminergic transporters for which it shows high affinity (Cui et al., 2009; Martí et al., 2017). In dopaminergic neurons, MPP+ inhibits complex I of the mitochondrial electron transport chain, causing mitochondrial dysfunction as well as increased oxidative stress. This damage eventually leads to apoptosis and necrosis of the neurons (Nicklas et al., 1985; Mizuno et al., 1987). These findings are underpinned by the results of RNA sequencing analyses. The genes involved in oxidative phosphorylation (*Atp6ap1l, Atp6v1e1*, and *Ndufa7*), the apoptosis (*Lrrc 74b, Lrrc 18*, and et al.), and the necroptosis (*Hist2h2aa2, Zbp1*, and *Fam47e*) were differentially expressed in the MPTP-induced PD mouse model (Yang et al., 2020). The severe impairment of dopaminergic neurons subsequently results in PD-like motor symptoms (Sayre et al., 1989).

Many studies have been conducted using MPTP-induced PD models in different species of experimental animals. Among them, the monkey PD model is currently the gold standard for preclinical testing of therapies (Bezard and Przedborski, 2011). Meanwhile, numerous studies have used this model in mice (Przedborski et al., 2001). In general, PD-like symptoms and a significant decrease in striatal dopamine are more satisfactorily achieved in the monkey model than in mice. The satisfying reproducibility of the MPTP-induced PD symptoms, pathological changes, and mitochondrial dysfunction in primates makes it the most valuable and practical model for PD studies at present.

One of the earliest studies demonstrating ferroptosis in PD model animals came from experiments using the MPTP model (Do Van et al., 2016). In mice administered MPTP, a loss of TH-positive neurons was observed in both the substantia nigra and striatum and these losses were prevented by the pre-administration of Fer-1 (Do Van et al., 2016). The same authors also demonstrated that MPP+ induced ferroptosis in Lund human mesencephalic (LUHMES) cells, which are immortalized dopaminergic neuronal precursor cells that can differentiate into dopaminergic neurons (Zhang et al., 2014). Moreover, they observed Fer-1 and/or deferiprone (DFP) suppressible cell death in LUHMES cells by PQ, rotenone, 6-OHDA, or MPP+ (Do Van et al., 2016). They also reported that SH-SY5Y cells are resistant to a ferroptosis inducer (erastin) while highly sensitive to an apoptosis inducer (staurosporine; Do Van et al., 2016). Despite the tendency towards apoptosis, Ito et al. demonstrated that MPP+-induced death of SH-SY5Y neuronal cells can be partially inhibited by both ferrostatin-1 and necrostatin-1 (Ito et al., 2017).

## Concluding Remarks

Although several mechanisms and cell death pathways are assumed to contribute to the neuronal death in PD, researchers have not conclusively established which might be the primary and most significant mechanism, or if there is a multifactorial cascade for PD pathogenesis. Recently, it was reported that synuclein aggregation can induce ferroptosis by interacting with membranes and accelerating lipid peroxidation (Angelova et al., 2020). Since synuclein is one of the causative genes of familial PD and a major component of Lewy bodies, this report strongly suggests the role of ferroptosis in PD pathogenesis. PD is also characterized by a decrease in dopamine levels in the substantia nigra and subsequent loss of dopaminergic neurons. Since dopamine itself seems to have anti-ferroptotic properties (Wang et al., 2016), the decreased dopamine levels in PD might render the cells highly susceptible to ferroptosis. Given the importance of synuclein as well as dopamine, LUHMES cells might be a better choice than SH-SY5Y cells for the study of ferroptosis in PD models. The reason is as follows: (1) SH-SY5Y cells express none or trace levels of synuclein. Many PD studies using SH-SY5Y cells were performed after establishing stable cell lines expressing exogenous synuclein. In contrast, LUHMES cells express a substantial amount of synuclein during differentiation into post-mitotic DA neurons by use of tetracycline (to switch-off the tetracycline-responsible *myc* gene), cyclic AMP, and GDNF (Lotharius et al., 2002, 2005); (2) although SH-SY5Y cells show features of DA neurons, they also show features of NAergic (noradrenergic) neurons (Filograna et al., 2015). Indeed, as mentioned above, Do Van et al. (2016) observed ferroptosis in LUHMES cells treated with a panel of chemical PD inducers. These difficulties regarding the choice of appropriate cells to examine ferroptosis in chemical PD models may be reduced by a recent report showing a method to evaluate the susceptibility of cells to ferroptosis; cellular NADPH level is proposed as a predictor for ferroptosis susceptibility (Shimada et al., 2016). However, whether ferroptosis truly contributes to the pathogenesis of PD or not remains a problem currently being studied. One of the difficulties of the ferroptosis study is the examination criteria of ferroptosis; lipid peroxidation, reliability of iron, and effectiveness of ROS scavengers as well as iron chelators are the criteria by which we can discriminate ferroptosis from other cell deaths. These parameters were especially difficult to examine in tissue contexts. To overcome this obstacle, Feng et al. (2020) screened a pool of antibodies and found several antibodies that stain cells undergoing ferroptosis in tissue sections. These antibodies include anti-TfR1, anti-malondialdehyde adduct, and anti-4-hydroxynonenal (4HNE) antibodies (Feng et al., 2020). Thus, the true contribution of ferroptosis in PD pathology should be unveiled in the near feature.

## Author Contributions

TA oversaw the formation of the whole manuscript. SW and TA wrote the manuscript. KUn and KUe contributed to the editing and proofreading of the manuscript. All authors contributed to the article and approved the submitted version.

## Conflict of Interest

One of the authors (SW) receives a scholarship funded by the Cooperation Program between TMDU and Sony IP&S, Inc. The funder was not involved in the study design, collection, analysis, interpretation of data, the writing of this article or the decision to submit it for publication. The remaining authors declare that the research was conducted in the absence of any commercial or financial relationships that could be construed as a potential conflict of interest.
